# Denosumab-induced hypocalcemia in patients treated with dialysis: an avoidable complication?

**DOI:** 10.1093/ckj/sfae048

**Published:** 2024-03-13

**Authors:** Maria Jesús Lloret, Hanne Skou Jørgensen, Pieter Evenepoel

**Affiliations:** Department of Nephrology, Fundació Puigvert, Barcelona, Spain; Institut de Recerca Sant-Pau (IR-Sant Pau), Barcelona, Spain; Institute of Clinical Medicine, Aarhus University, Aarhus, Denmark; Department of Nephrology, Aalborg University Hospital, Aalborg, Denmark; Department of Microbiology, Immunology and Transplantation, Nephrology and Renal Transplantation Research Group, KU Leuven, Leuven, Belgium; Department of Microbiology, Immunology and Transplantation, Nephrology and Renal Transplantation Research Group, KU Leuven, Leuven, Belgium; Department of Nephrology and Renal Transplantation, University Hospitals Leuven, Leuven, Belgium

Patients with chronic kidney disease (CKD) experience a fracture risk that is several-fold higher than the background population, but the treatment of these patients is challenging due to concomitant mineral and bone disorder (CKD-MBD) [1]. Antiresorptive therapy is the first-line therapy for fracture prevention in the general population, with denosumab rapidly gaining interest in the nephrology community because of its potency and non-renal clearance. With increased use in patients receiving dialysis, safety concerns have emerged, in particular the risk of hypocalcemia. In a recent JAMA paper, Bird and colleagues [2] reported a 12-week cumulative incidence of severe hypocalcemia (defined as albumin-corrected calcium <7.5 mg/dL) as high as 41% in patients on dialysis treated with denosumab *vs* 2% with oral bisphosphonates. Life-threatening symptoms (seizures, arrhythmias) occurred in 5.4% of denosumab-treated patients and 1.3% died. These figures, though not surprising to most nephrologists, call for reflection and action.

Remember, CKD-MBD first: Bird *et al*. rightly conclude that denosumab should only be administered after careful patient selection and with a plan for frequent monitoring [2]. Management of osteoporosis for patients on dialysis should be centred in the dialysis unit because it needs to be integrated in the overall treatment of CKD-MBD [1]. Knowledge of bone turnover determines therapy choices in this patient population [3]. A high bone turnover calls for parathyroid hormone (PTH) suppressive therapy, particularly if the plan is to initiate (potent) antiresorptive therapy. High turnover bone disease is a robust risk factor for iatrogenic hypocalcemia, as PTH suppression (by parathyroidectomy, calcimimetics) or antiresorptive therapy will induce a state of hungry bone. Coordination of care in the dialysis unit should also include a post-denosumab hypocalcemia prevention regimen, which may involve (transient) increase of the calcium supply via diet, supplements or dialysate, ensuring adequate intake of vitamin D and close monitoring of serum calcium levels.

**Figure 1:**
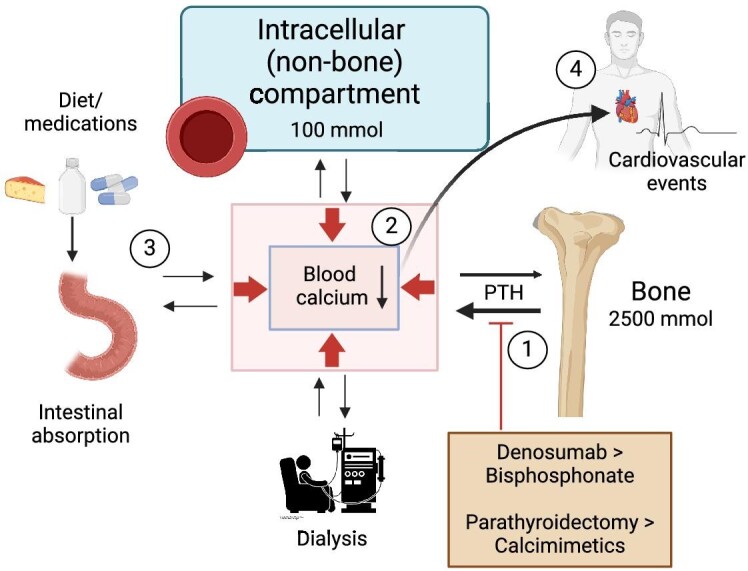
(**1**) Potent antiresorptive or PTH suppressive therapy reduce skeletal calcium efflux, resulting in (**2**) hypocalcemia. Awareness and implementation of a hypocalcemia prevention program with increased calcium supply (**3**) and close monitoring may alleviate iatrogenic hypocalcemia and related health risks (**4**). Created with BioRender.com.

Feed the hungry bone: the high incidence of post-denosumab hypocalcemia in late-stage CKD unmasks a situation where serum calcium levels are dependent on ongoing bone resorption (Fig. 1). In other words, bone has become a main source of calcium for the patient. This notion is supported by calcium isotope studies documenting skeletal calcium efflux in dialysis patients on a regular diet, even when treated with calcium supplements [4]. A recent consensus statement by the European Renal Association and the European Society of Paediatric Nephrology recommends a daily calcium intake of 800–1000 mg in patients with CKD to maintain neutral calcium balance [5]. In severe hyperparathyroidism, bone is in a calcium deficit and will rapidly re-mineralize when PTH levels decrease. A temporary higher calcium supply is therefore often appropriate following PTH suppressive or antiresorptive therapy [5].

Go and preach: post-denosumab hypocalcemia remains unnoticed by most prescribers. Bird and colleagues observed that denosumab was rarely prescribed by nephrologists (2.3%) [2]. Nephrologist should be more active not only in promoting anti-fracture treatment, but also in informing primary care clinicians and osteoporosis experts about its inherent risks [6]. Denosumab is a true asset in tackling osteoporosis, including for patients with CKD; however, prescribers need to be cognizant of the risks involved.
